# Measuring two-photon microscopy ultrafast laser pulse duration at the sample plane using time-correlated single-photon counting

**DOI:** 10.1117/1.JBO.25.1.014516

**Published:** 2020-01-29

**Authors:** Youngchan Kim, Steven S. Vogel

**Affiliations:** U.S. National Institutes of Health, National Institute on Alcohol Abuse and Alcoholism, Section on Cellular Biophotonics, Laboratory of Molecular Physiology, Bethesda, Maryland, United States

**Keywords:** multiphoton microscopy, time-correlated single-photon counting, second-harmonic generation, pulse duration, interferometric autocorrelation

## Abstract

Two-photon microscopy (2PM) has revolutionized biomedical imaging by allowing thin optical sectioning in relatively thick biological specimens. Because dispersive microscope components in 2PM, such as objective lens, can alter temporal laser pulse width (typically being broader at the sample plane), for accurate measurements of two-photon absorption properties, it is important to characterize pulse duration at the sample plane. We present a simple modification to a two-photon microscope light path that allows for second-harmonic-generation-based interferometric autocorrelation measurements to characterize ultrafast laser pulse duration at the sample plane using time-correlated single-photon counting (TCSPC). We show that TCSPC can be used as a simple and versatile method to estimate the zero time delay step value between two adjacent ultrafast laser pulses for these measurements. To demonstrate the utility of this modification, we measured the Coherent Chameleon-Ultra II Ti:sapphire laser pulse width at the sample plane using a 10× air, 40× air, or 63× water-immersion objective lens. At 950-nm two-photon excitation, the measured pulse width was 154±32, 165±13, and 218±27  fs (n=6, mean±standard deviation), respectively.

## 1 Introduction

Over the past three decades, since two-photon microscopy (2PM) was first demonstrated by Denk et al.,^1^ applications of 2PM have significantly contributed to our knowledge of a broad range of biological phenomena by allowing noninvasive imaging of biological specimens in three dimensions.^2^^–^^4^ Furthermore, since an ultrafast pulsed-laser source for two-photon excitation generates an inherently small excitation/observation volume (∼subfemtoliter), 2PM has played a pivotal role in introducing powerful time-correlated single-photon counting (TCSPC) techniques, such as fluorescence lifetime imaging (FLIM) and fluorescence correlation spectroscopy to the biomedical community.^5^^–^^7^

The efficacy of 2P excitation is dependent on the laser wavelength, its average power, repetition rate, and pulse width. While wavelength, power, and repetition rate are readily measured using standard laboratory equipment, ultrafast laser pulse width, usually ranging between 70 and a few hundred femtoseconds in duration, can be measured using highly specialized equipment. Furthermore, commercial autocorrelators used to measure pulse width are expensive and typically measure the pulse width as the laser beam leaves the laser, not at the sample plane of a two-photon microscope. Group velocity dispersion (due to microscope dispersive optical components) can alter pulse width, which is, characteristically, broader at the sample plane.^8^^–^^11^

In this paper, we present a second-harmonic generation (SHG)-based interferometric autocorrelation technique to characterize ultrafast laser pulse duration at the sample plane using a simple modification to a two-photon microscope light path. We demonstrate that TCSPC can be used as a simple and versatile method to estimate the zero time delay step value between two adjacent ultrafast laser pulses for these measurements. Finally, we demonstrate the utility of this approach by measuring the laser pulse width at the sample plane after passing through three different objective lenses.

## 2 Methods and Materials

### 2.1 Experimental Setup

**Fig. 1:**
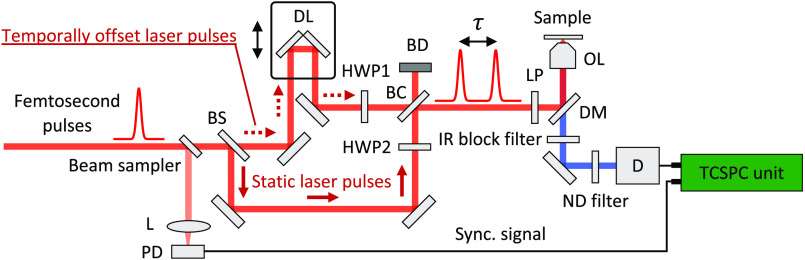
Schematic diagram of a modification to a two-photon excitation time-resolved fluorescence microscopy to allow characterizing ultrafast laser pulse duration at the sample plane. The dotted and solid red arrows indicate the directions of the temporally offset and static laser pulses, respectively. BS, beam splitter; HWP, half-wave plate; L, lens; LP, linear polarizer; DL, delay line; D, hybrid detector; PD, photodiode detector; BC, beam combiner; OL, objective lens; DM, dichroic mirror; BD, beam dump.

The experimental setup for *in situ* measurement of femtosecond laser pulse duration is illustrated in Fig. 1. A mode-locked Ti:sapphire laser (Chameleon-Ultra II, Coherent, Santa Clara, California) operating at 80-MHz repetition rate and 950-nm center wavelength was used as an excitation source for an inverted microscope (Axio Observer D1; ZEISS, Oberkochen, Germany). The linearly polarized laser beam was first spatially filtered and expanded (not shown in diagram) by a spatial filter assembly (KT310; Thorlabs, Newton, New Jersey). A small part of the main beam picked up through a wedged beam sampler (10B20-01NC.2; Newport, Irvine, California) was used to synchronize laser pulse excitation with data acquisition using a fast photodiode detector (DET10N; Thorlabs). The laser beam was split into two arms via a 50:50 beam splitter (UFBS5050; Thorlabs). After passing through each half-wave plate (AHWP10M-980; Thorlabs), the two beams were subsequently recombined via a second 50:50 beam splitter (UFBS5050; Thorlabs). The relative time delay τ between two adjacent pulses was controlled using the motorized delay line (ILS200CC; Newport), with 200-mm travel range (corresponding to 1.333-ns optical delay). The recombined beam passed through a linear polarizer (LPNIR100-MP; Thorlabs), so that the intensity of each laser pulse could be independently adjusted by rotating its specific half-wave plate, and also to confirm that each laser pulse has the same linear polarization at the sample plane. A multiphoton short-pass dichroic beam splitter (FF670-SDi01-25636; Semrock, Rochester, New York) was used to reflect the excitation beam to an air microscope objective (Zeiss 40×/0.9  NA, Jena, Germany). The emission from the sample was collected by the same objective lens and spectrally filtered by transmitting through the same dichroic beam splitter. An infrared block filter (BG39; SCHOTT, Mainz, Germany) located immediately after the dichroic beam splitter was used to block any residual two-photon excitation. Additional neutral density filter located in front of the detector was included to adjust SHG signal intensity. A hybrid detector (HPM-100-40; Becker & Hickl, Berlin, Germany) connected to a detector control module (DCC-100; Becker & Hickl) was used to detect the SHG signal. Detected photons were processed with a TCSPC module (SPC-150; Becker & Hickl). The dark count rate for the detector was 300 cps at room temperature. A custom-written LabVIEW program was used to control the motorized delay line and to provide a trigger signal to SPCM software (ver. 9.81; Becker & Hickl) running in single (SING) mode for data acquisition and storage.

### 2.2 Second-Harmonic Generation

Dry sodium phosphate monobasic monohydrate (${\mathrm{NaH}}_{2}{\mathrm{PO}}_{4}{\cdot H}_{2}O$; Mallinckrodt Baker, Inc., Paris, Kentucky) powder loaded into a glass-bottom dish (P35G-1.5-10-C; MatTek, Ashland, Massachusetts) was used to generate a 475-nm second-harmonic signal from the ultrafast Ti:sapphire laser. Second-harmonic signals were adjusted to yield a photon count rate between ∼10,000 and ∼100,000  cps to avoid both TCSPC time bin saturation and pile-up artifacts.^12^

### 2.3 Data Analysis

IGOR Pro 8 software was used to process and fit interferometric autocorrelation data. GraphPad Prism (ver. 8.2.1) was used to calculate mean, standard deviation (SD), analysis of variance, and the Kruskal–Wallis test with Dunn’s multiple comparisons.

## 3 Results

**Fig. 2:**
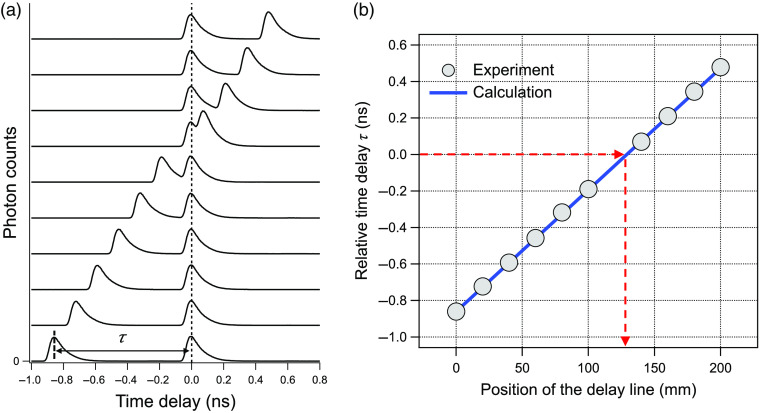
(a) SHG-based time-resolved traces with varying relative time delay τ between the static and temporally offset laser pulses. Each time-resolved SHG trace was offset vertically for clarity. The dotted line indicates zero time delay (τ=0), as well as the position of the static laser pulse pathway. (b) The measured and calculated relative time delay was plotted as a function of the position of the delay line ranging from 0 to 200 mm. The position of the delay line SHG pulse was used to calculate the relative time delay (blue trace). Through these TCSPC measurements, one can rapidly estimate the delay line position where both laser pulses overlap temporally as shown by the dotted red arrows.

Our light path modification generates a pair of ultrafast laser pulses with one pulse being static every 12.5 ns (for an 80-MHz laser) and the second pulse being temporally offset from the static pulse by between −860 and +479  ps. We first tested this method by measuring the time-resolved SHG traces generated from both static and temporally offset ultrafast pulses. By sweeping the delay line over the possible scanning range of 200 mm, it was observed that the relative time delay τ between the static and temporally offset laser pulses was shifted accordingly, as shown in Fig. 2(a). Figure 2(b) shows that the measured relative time delay was in good agreement with the calculation from the position of the delay line. Using this approach, one can also estimate the position of the delay line associated with zero time delay between the two laser pulses (τ=0). The “decay-like” feature observed in time-resolved SHG traces, shown in Fig. 2(a), is caused by a convolution with the instrument response function of our TCSPC system, and the full width at half maximum (FWHM) of an SHG signal from a SING ultrafast laser pulse can be used to estimate the instrument response function of the system, in this case, ∼95  ps.

**Fig. 3:**
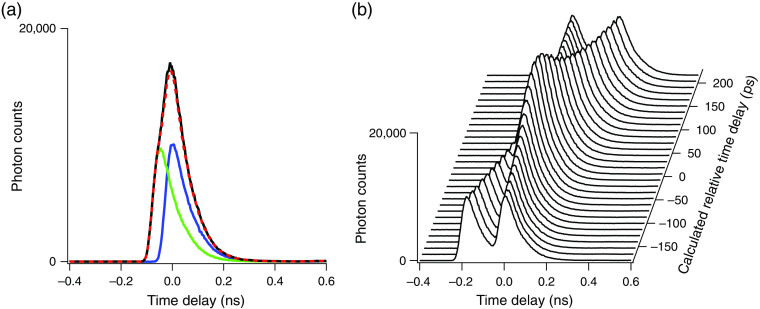
(a) Time-resolved SHG traces obtained when either the delay line optical pathway was blocked (blue), the static delay optical pathway was blocked (green), or without any blocking (black). Here, the relative time delay between the blue and green traces was 49 ps. The sum of the two individual SHG traces from SING laser pulses (green+blue) is plotted for comparison with the black trace (dotted red trace). With a 49-ps delay, the black trace cannot resolve the two individual laser pulses that form it. (b) Time-resolved SHG curves are plotted for varying τ values. The τ values ranging from −180 to 217 ps with 13-ps step-increases were used to illustrate the range of τ values where our microscope cannot resolve the two laser pulses (∼−100 to 100 ps).

As expected, when the absolute value of the relative time delay between the two laser pulses are less than or equal to the instrument response function, we could no longer resolve the two SHG traces as shown in Figs. 3(a) and 3(b). Interestingly, we did not observe any interferometric signal in Fig. 3(b). Presumably, the relatively large time delay steps used for these measurements (13 ps, corresponding to a 2-mm delay step size) jumped over the region where positive and negative interferences between the two laser pulses were expected to be observed.

**Fig. 4:**
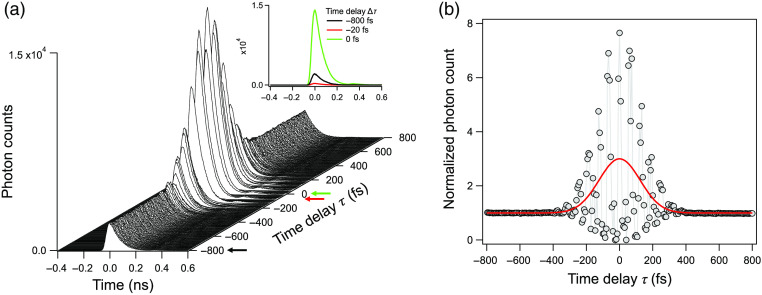
(a) Time-resolved SHG curves are plotted for varying τ values. The τ values ranging from −800 to 800 fs with 6.7-fs step-increases were used to illustrate the range of τ values (∼±300  fs) where the temporally overlaping laser pulses display both constructive and destructive interferences. Inset shows representative time-resolved SHG traces where constructive (green), destructive (red), and no (black) interferences were observed. The black, red, and green arrows on the time delay axis indicate time delays of −800, −20, and 0 fs between two adjacent ultrafast laser pulses, respectively. (b) The interferometric SHG autocorrelation trace (gray line and circle) for τ values ranging from −800 to 800 fs in 6.7-fs steps was measured through a ZEISS 63× water-immersion objective lens at the sample plane. The intensity is normalized to the average background. The solid red trace corresponds to a Gaussian fit of the intensity autocorrelation and is used to measure its FWHM, 291 fs and in turn this was then used to estimate the laser pulse duration assuming a ${\mathrm{sech}}^{2}$ pulse profile by multiplying the Gaussian fit FWHM by 0.648 to yield an estimated laser pulse width of 189 fs.

To observe these interreference patterns, we next scanned delay times bracketing our calculated τ=0 value with 6.7-fs steps (1-μm delay step) from −800 to 800 fs, as shown in Fig. 4(a). The green, red, and black traces in the inset of Fig. 4(a) correspond to the time-resolved SHG traces associated with constructive, destructive, and no interference between the static and temporally offset laser pulses, respectively. Thus, we conclude that the TCSPC data are useful to rapidly estimate the zero time delay position, so as to effectively characterize ultrafast pulse width. Figure 4(b) shows a typical interferometric autocorrelation trace ranging from −800 to 800 fs obtained at the sample plane using a 63×/1.2 water-immersion objective lens (Zeiss) by integrating photon counts over 12.5 ns for each time-resolved SHG curve in Fig. 4(a). The normalized SHG photon count was calculated by subtracting the minimum photon count from the raw trace and subsequently normalizing the average background photon count of the subtracted trace (where τ<−500  fs or >500  fs). Since the peak-to-background ratio was close to 8:1 (see Sec. 6) and we observed near-perfect symmetry of the interferometric autocorrelation around zero time delay, we concluded that the experimental setup was optically well-aligned. The normalized interference trace was fit to a Gaussian function to measure the FWHM, i.e., 291 fs, corresponding to an effective pulse duration of 189 fs assuming an unchirped ${\mathrm{sech}}^{2}$-shape pulse profile.^13^ Since the interferometric autocorrelations measured by this approach are obtained without phase stabilization, reproducible interferometric phase information is hard to measure. For this reason, as shown in Fig. 4, the interferometric autocorrelation trace was fit using a simple Gaussian model to estimate the intensity autocorrelation.

To demonstrate the utility of this method, we measured FWHM pulse durations at the sample plane through three different objectives: 10×/0.3 EC Plan-Neofluar, 40×/0.9 EC Plan-Neofluar, and 63×/1.2W C-Apochromat (Zeiss). The mean and SD values from six measurements for the 10×, 40×, and 63× objective lenses were 154±32, 165±13, and 218±27  fs, respectively. These results indicate that the pulse duration values for the 10× and 40× objective lenses were significantly shorter than the 63× objective lens (p<0.01, by analysis of variance), indicating that the 63× objective lens induces more pulse broadening than the 10× or 40× objective lenses. Presumably, the additional glass components required for chromatic and spherical aberration correction in an apochromatic lens result in pulse broadening. A direct comparison of the efficacy of different objectives for the two-photon excitation is complicated because in addition to supporting different pulse durations, as we have demonstrated here, different objectives may also have different numerical apertures, spectral characteristics, and point spread functions. If these variables are not altered, and assuming there is no saturation of the fluorescent signal, two-photon intensity has been shown to be inversely proportional to the excitation pulse width.^14^

## 4 Discussion

**Fig. 5:**
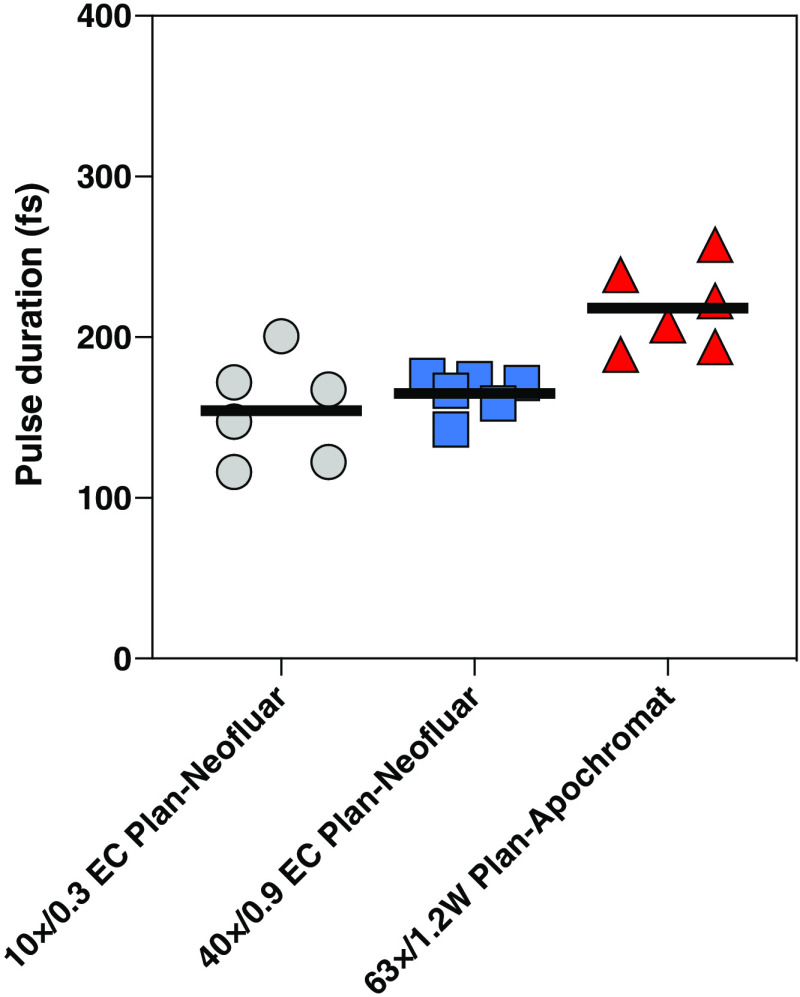
The FWHM pulse durations of the femtosecond laser pulse were measured at the sample plane through 10× (gray circle), 40× (blue square), and 63× (red triangle) microscope objective lenses. Bars indicate the mean from six individual measurements.

To circumvent the difficulties for characterizing ultrafast pulse duration at the sample plane, we developed and tested a simple and reliable method for interferometric autocorrelation measurements using TCSPC. Because the mean pulse duration values for the 10×, 40×, and 63× objective lenses were measured, as shown in Fig. 5, we can estimate total group delay dispersion, D in ${\mathrm{fs}}^{2}$, of each microscope system using the following equation:^3^^,^^15^^,^^16^

$$D=\tau_{\text{in}}^{2}\sqrt{\frac{{\left(\tau_{\text{out}}/\tau_{\text{in}}\right)}^{2}-1}{7.68},}$$ (1)

where $\tau_{\text{in}}$ and $\tau_{\text{out}}$ are FWHM of the excitation pulse before and after dispersive microscope optical components, respectively. Assuming 140 fs for the pulse duration before the dispersive components (based on the manufacturer’s laser specification), the total group delay dispersion at 950 nm for each objective lens corresponds to 3241, 4411, and $8442\;{\mathrm{fs}}^{2}$, respectively. These values are in good agreement with a typical range of total group delay dispersion in 2PM (between 3000 and $20,000\;{\mathrm{fs}}^{2}$).^3^ It is noteworthy that the efficacy of two-photon excitation fluorescence is inversely proportional to the temporal pulse width of the excitation pulse.^3^^,^^15^ Thus, the method described here can be used to measure the altered pulse duration at the sample plane, and along with wavelength, optical power, and repetition rate of the two-photon excitation laser, these values can be used and adjusted to maintain the same level of two-photon fluorescence intensity.

Several interferometric measurement approaches have been used to measure laser pulse width at the sample plane of a multiphoton microscope.^8^^,^^10^^,^^11^^,^^17^^–^^20^ What is unique to the approach presented here is the use of TCSPC. In comparison to simple intensity measurements used with the other approaches, TCSPC records photon counts as a function of time with high precision and accuracy, as illustrated in Figs. 2–4. This capability has great utility for determining the exact delay stage position where the two laser pulses, as used for autocorrelation measurements, overlap in time (zero time delay), as illustrated in Fig. 2. A negative aspect of this approach is that it requires the added expense of acquiring a TCSPC module (∼$13,000), a component that is not absolutely required for autocorrelation interferometric measurements. Nonetheless, we believe that this added expense is warranted when factoring in the time and effort saved in aligning a microscope-based interferometer. We also note that many multiphoton systems already have TCSPC modules for multiphoton FLIM and that the expense of purchasing a TCSPC module is a relatively small fraction of the expense involved in building or buying a multiphoton microscope.

Other differences between our approach to measuring laser pulse width at the sample plane and approaches used in some of the previously reported methods^8^^,^^10^^,^^11^^,^^17^^–^^20^ involve: (1) the method used for scanning the delay line laser pulse and (2) how multiphoton excitation is detected. In reference to the first point, in our system, we use a motorized delay line with a 200-mm travel range in 1-μm discrete steps, which corresponds to 1.333-ns maximal optical delay in 6.7-fs steps. Delay lines with a shorter step size (0.1  μm) are also available and their use would allow 0.67-fs steps in our system. This in turn would allow higher precision for measuring interferometric fringes in the autocorrelation function but was not deemed necessary in this study. One of the advantages for using a long-travel motorized delay line in conjunction with an interferometric setup is that these same components can also be used as the basis for building an ultrafast pump–probe spectroscopy setup. Other approaches for measuring laser pulse width have used continuous scanning methods, such as vibrating mirrors mounted on a modulated speaker or motor to continuously scan the delay line. These systems are typically less expensive to implement but have a much shorter travel range and therefore require more precise alignment. In reference to the second point, in our system, ultrafast infrared laser pulses are converted to visible light using SHG and are detected using the same hybrid detectors we use for multiphoton microscopy. Other approaches have utilized either a dedicated photodiode detector to record two-photon-induced conductivity^19^^,^^20^ or a fluorescent plate–PMT (photomultiplier tube) detector combination for recording two-photon-excited fluorescence. We used SHG for this purpose in our application because it did not require a dedicated diode detector and because SHG has several advantages over using a dye in this application. SHG does not have an excited singlet state (such as fluorescence) and therefore does not have a risk of entering into a triplet state, which can result in a low quantum yield as compared to an SHG substrate. Furthermore, the dry ${\mathrm{NaH}}_{2}{\mathrm{PO}}_{4}{\cdot H}_{2}O$ powder that we use as an SHG substrate is less expensive than most multiphoton dyes, does not noticeably degrade with time, and therefore does not need to be prepared fresh before use. Finally, it has been brought to our attention that a commercial autocorrelation system (Carpe, APE, Berlin, Germany) can measure pulse width at the sample plane of a microscope. This dedicated instrument employs a vibrating mirror in the delay path with a maximal time delay window of 15 ps.

## 5 Conclusion

In conclusion, we have presented a method for observing and analyzing interferometric autocorrelation measurements of ultrafast laser pulses at the sample plane using the TCSPC technique. The proposed method has been shown to be able to estimate the zero time delay step value for these measurements by utilizing the TCSPC technique; this then allows direct access to the time-resolved SHG traces that interfere with each other. The interferometric autocorrelation measured at the sample plane enables us to estimate the ultrafast laser pulse duration. Furthermore, our measurements suggest that the 63× objective lens led to more temporal pulse broadening than the 10× or 40× objective lens. Because this broadening induces altered two-photon absorption properties, this change should be taken into account when selecting objectives for 2PM. We anticipate that this method may be very useful in studying multiphoton absorption properties.

## 6 Appendix A: Analytical Description of Interferometric Autocorrelation

The interferometric autocorrelation, also referred to as the second-order interferometric autocorrelation, was introduced for characterizing ultrafast optical pulses by Diels et al.^13^ In this process, the input beam is split into two identical parts in which E(t−τ) is delayed with respect to E(t) by a delay time τ. These two parts are subsequently combined and colinearly sent into a nonlinear crystal to generate the second-harmonic light of the interference pattern produced by the combined pulse sequence. With an assumption of a Gaussian beam profile, the electric field of the laser pulses can be described as

$$E(t)=E_{0}(t)e^{-\frac{t^{2}}{a^{2}}+i\omega t},$$ (2)

where ω is the carrier frequency of the electric field and a is the radius in which the amplitude of the electric field is 1/e of its maximum amplitude. The equation describing the interferometric autocorrelation with E(t) given by Eq. (2) is

$$I(\tau )=\overset{\infty }{\underset{-\infty }{\int }}{\left|{\left[E(t)+E(t-\tau )\right]}^{2}\right|}^{2}dt,$$ (3)

$$I(\tau )\propto 1+2e^{-\frac{\tau^{2}}{a^{2}}}+4e^{-\frac{3\tau^{2}}{4a^{2}}}\;\cos(\omega \tau )+e^{-\frac{\tau^{2}}{a^{2}}}\;\cos(2\omega \tau ).$$ (4)
